# Class and objectification: An investigation into the relationship between women’s social class and self- and other-objectification

**DOI:** 10.1371/journal.pone.0214111

**Published:** 2019-04-30

**Authors:** Qian Ma, Steve Loughnan

**Affiliations:** Department of Psychology, School of Philosophy, Psychology and Language Studies, University of Edinburgh, Edinburgh, United Kingdom; Fordham University, UNITED STATES

## Abstract

This paper aims to investigate the relationship between women’s social class and their levels of self- and other-objectification. Two online studies comprising of multiple questionnaires were conducted: Study 1 examined the association between the social class (both objective and subjective) of 198 non-student British women and their self-objectification levels, while Study 2 turned towards the relationship between social class and other-objectification among 82 non-student British women. Our results indicated no apparent relationship between social class and each of the relevant objectification measures. As the first paper on the topic of class and objectification, it appears that there is no relationship observed between the two.

## Introduction

Objectification Theory was first proposed by Fredrickson and Roberts [1] as a framework to better understand women’s objectification experiences and their gender-role socialization and how these could further influence their wellbeing. More specifically, the theory proposed that exposure to sexual objectification tends to socialize women to view and value themselves based on how their bodies look to men. In this process, women internalize observers’ perspectives of their bodies through self-objectification which manifests itself as body surveillance—paying habitual attention to how their bodies look to other people. This self-objectification renders them more vulnerable to body/weight dissatisfaction, thereby contributing to the risk of multiple psychological problems such as increased body shame and appearance anxiety, reduced self-esteem, flow experiences, internal bodily awareness (e.g., hunger), eating disorders, sexual dysfunction, and depression [1, 2].

In addition, on an operational level, self-objectification is often measured by the participants’ self-reported levels of self-objectification or body surveillance [2]. The most commonly used questionnaires are the Self-Objectification Questionnaire (SOQ) [3] and the Body Surveillance subscale from the Objectified Body Consciousness scale (OBC) [2, 4, 5, 6]. In line with previous research, our research will employ both to measure self-objectification.

### Self-Objectification and social class

Despite the recognition of the negative impact of self-objectification, it is important to explore further whether this impact falls equally on all women. Most previous research in this area has focused on differences in age, sexual orientation, ethnicity, and nationality. A negative correlation has been found between age and each of the following variables: self-objectification, body surveillance, body shame, appearance anxiety, and dieting behavior [7, 8, 9]. Mixed findings have been detected for women with different sexual orientations. One study suggested that lesbian university students engaged in higher levels of body surveillance than heterosexual female students [10]. However, another study found that heterosexual women paid more attention to their physical attractiveness, were more dissatisfied with their bodies, and had a higher risk of developing eating disorders than lesbians [11]. Self-objectification among Caucasians, Hispanics, African Americans, and Asian Americans has been explored by Hebl, King, and Lin [12], who found that the level of self-objectification was highest among Hispanic individuals and lowest among African Americans. This paper importantly shows the power of socio-demographic variables in self-objectification. In considering self-objectification levels within different countries, an international study of seven countries found that women in India, Japan, and Pakistan reported lower self-objectification levels than those in Australia, Italy, the UK, and the US [13]. Moreover, another study has shown that compared with women from the US, women from Nepal engaged in less body surveillance [9]. In short, the socio-demographic characteristics of a woman can qualify the extent to which she self-objectifies.

Despite this recognition of the important role that socio-demographics can play in relation to variations in women’s self-objectification levels, no study has directly examined the association between social class, and self-objectification and body surveillance. Part of the reason may be that unlike age, sexual orientation, ethnicity, or nationality, identifying individuals’ social class is more methodologically complex. In social psychology, social class is commonly defined through two components: objective and subjective social class [14]. Objective social class is most commonly assessed by individuals’ education, occupational status, and income whereas subjective social class refers to individuals’ perception of which social class they belong to when compared to others [14]. Whether either or both are related to objectification is an open question.

Previous studies have investigated the relationship between social class and some potential outcomes of self-objectification (body/weight dissatisfaction and dieting behavior). McLaren and Kuhb [15] found that middle-aged women from upper class backgrounds experienced higher levels of body dissatisfaction than women of the same age from lower class backgrounds; in particular, higher educational attainment was associated with greater dissatisfaction with weight and appearance. Another study found that ethnic minority girls from lower income groups had greater levels of body satisfaction than minority girls from higher income groups [16]. Also, Kashubeck-West and Huang [17] indicated that studies showed women from an upper social class engaged in more dieting behavior. Similar results were observed across various countries. For instance, in a study on female university students in Turkey [18], higher social class was a predictor of greater weight dissatisfaction and more extreme dieting behavior. Therefore, although a direct link to self-objectification is lacking, class appears robustly linked to some outcomes of self-objectification—body dissatisfaction and extreme dieting behavior.

One study directly investigated income (one aspect of social class) and body surveillance [19], but its sample was solely comprised of participants from low income groups and therefore it was unable to robustly examine the effects of social class on body surveillance. In the absence of direct evidence, we conducted a simple secondary data analysis from another study. We compared the sample means of body surveillance ratings from two sets of female respondents that have been reported in this study [20]: one set from a private university (University of Delaware) (*N* = 161, *M* = 4.65, *SD* = 1.07) and the other from a public university (Arizona State University (*N* = 202, *M* = 3.28, *SD* = 0.78); we found a significant difference between the two groups, t (361) = 14.10, *p* < 0.001. If the substantial difference in tuition fees between the two universities at least partially reflects social class differences between their student populations, this could imply, and it would be reasonable to expect, that female students who come from a higher family income background would experience higher levels of body surveillance. However, as income is just one aspect of social class, and it has not been directly measured in this study, further research is needed to provide a more accurate measure of social class.

This paper aims to address the paucity of direct studies on the relationship between social class and women’s self-objectification levels. Again, it is important to explore whether and how self-objectification falls equally or differently on all women as previous research has been limited to investigating the role of age, sexual orientation, ethnicity, and nationality. Our research focuses on whether there is a class-based impact: if higher social class is associated with increased self-objectification, it is a previously unrecognized detriment to increased social class. Generally, it is beneficial to raise one’s social class [21]; however, if increased self-objectification is a widespread experience of upper class women, this may be an example of where this assumption breaks down. Society is increasingly polarized, with rising levels of inequality [22], and we can therefore expect class to play a more central role in people’s lives. If our research shows certain social classes are more susceptible to self-objectification this can spur future research to determine the reasons why and ultimately seek to mitigate any potential negative effects heightened self-objectification may have on women’s health.

In addition, as most of the existing research on self-objectification and body surveillance has been based on a narrow convenience sample of university students [2], this paper will shift its sample to other groups of women (non-student females). Study 1 hypothesizes a positive association between women’s social class and their self-objectification levels, with upper class women predicted to engage in more self-objectification and body surveillance, and lower class women predicted to engage in less self-objectification and body surveillance.

### Other-objectification and social class

Previous research has shown that not only do women objectify themselves, but they also objectify other people: more specifically, women with higher self-objectification levels tend to exhibit greater objectification of other women and men [23]. Therefore, if social class has an impact on self-objectification, it may also influence other-objectification. As with the lack of studies on women’s social class and self-objectification, research on the relationship between women’s social class and their objectification of others is similarly limited.

Other-objectification refers to viewing a person as an object and denying his or her humanity. It involves the process of dehumanization, which refers to “decreased attributions of human nature, competence, warmth, and morality” [24]. As such, researchers often consider dehumanization to be an indicator of objectification, and many studies have been conducted regarding the dehumanization of others. Heflick and Goldenberg [25] found that when individuals concentrated on a woman’s appearance, they were more likely to perceive her as having fewer human characteristics. Moreover, other studies in multiple nations have suggested that compared with females who are fully clothed, sexualized females were more likely to be dehumanized—perceived to have less intelligence, agency, humanness, and morality [26, 24]. Together, the above studies have reinforced the notion that objectified females were viewed as having fewer human characteristics. Further research has shown that in addition to sexualized females, sexualized males were also perceived to be less human in terms of having fewer mind attributions and less morality [24, 13].

Instead of assessing whether participants dehumanized sexualized targets, many previous studies examined participants’ attitudes towards a variety of factors and focused on whether participants associated any specific groups of people with having fewer human characteristics. According to Haslam [27], dehumanization was often linked to stereotypes involving minority groups; for example, African Americans were more likely to be denied their human nature and more frequently compared with apes than other racial groups [27]. Likewise, people with disabilities have historically been belittled as “parasites that infect the social body” [27]. Among non-minority groups, sexually mature women tended to be dehumanized more than non-sexually mature girls [1]. When considering social class, Loughnan, Haslam, Sutton, and Spencer [28] found people tended to assign individuals from low socioeconomic backgrounds a broader animality that denied them their human characteristics.

However, the above research only focused on the extent of dehumanization and other-objectification as experienced by different target groups. Very few studies have focused on the perceivers or considered whether people from different groups objectify and dehumanize others differently. In particular, we are aware of no studies in this area which examine the impact of social class on perceivers and whether it affects how they objectify/dehumanize other people. Therefore, the aim of Study 2 is to investigate whether there are any differences on the perceivers’ side and in particular, whether there is a relation between women’s social class and how women objectify and dehumanize others. However, due to the lack of research in both the self- and other-objectification fields, and without the results from Study 1, we will leave Study 2 as an exploratory hypothesis–to find out whether there is a relationship between women’s social class and their other-objectification level and if so, explore the relationship.

## Study 1

### Methods

This study was approved by the Psychology Research Ethics Committee, School of Philosophy, Psychology and Language Sciences, the University of Edinburgh.

#### Participants

204 British women were recruited online from Prolific Academic. Each participant was paid £1, and 198 sets of responses were used, with the remaining six excluded due to incomplete responding. All participants were non-students aged between 18 and 71 years old (*M* = 38.67, *SD* = 11.18), and 92.4% were white (*N* = 183).

#### Measures

All materials and data can be found online (osf.io/9e2qp). An online questionnaire comprising of four surveys examined the association between women’s social class and their self-objectification levels:

- A demographic questionnaire to capture basic information including age, ethnicity, and marital status.
- The MacArthur Sociodemographic Questionnaire (MSQ) [29] to measure both subjective and objective social class. Objective social class was combined and assessed through participants’ current education, occupation, and income; however, because the MSQ was created in the US, questions on education and income were edited in order to suit the British context. In addition, participants’ own annual income (before tax) was measured using nine income brackets ranging from “below £5000” to “£150,000 and above”. Participants’ highest educational attainment was measured through eight categories ranging from “lower than high school qualification” to “professional (MD, JD, DDS, etc.)”. Occupation was measured in two parts, with participants asked to fill in both the kind of business or industry in which they work or worked (for example, healthcare) and their job title (for example, registered nurse). Using Chan and Goldthorpe [30]’s status groups, each participant’s occupation was first assigned into one of 31 broader occupational categories. These categories were then divided into 8 status bands (a new band was added for unemployed participants), with each band given a corresponding score between 0 and 7:
  Status band i—higher managers and professionals (7 points)
  Status band ii—lower managers and professionals (6 points)
  Status band iii—intermediate employees (5 points)
  Status band iv—small employers and own-account workers (4 points)
  Status band v—lower supervisors and technicians (3 points)
  Status band vi—semi-routine workers (2 points)
  Status band vii—routine workers (1 point)
  Status band viii—unemployed (0 points)

Data on participants’ highest education level, occupation, and income has been compiled in Table 1.

**Table 1:** Social class data of sample (*N* = 198).

Variables	% (n)
**Occupation**	
Unemployed	2.5 (5)
Routine workers	10.1 (20)
Semi-routine workers	14.6 (29)
Lower supervisors and technicians	6.1 (12)
Small employers and own-account workers	9.1 (18)
Intermediate employees	31.3 (62)
Lower managers and professionals	12.6 (25)
Higher managers and professionals	12.1 (24)
**Education**	
Lower than high school qualification	2.5 (5)
High school/secondary school qualification	26.3 (52)
Associate degree/vocational qualification	10.6 (21)
Bachelor's degree/undergraduate Master’s degree	42.9 (85)
Postgraduate Master's degree	12.6(25)
Doctorate	2.0 (4)
Professional (MD, JD, DDS, etc.)	2.5 (5)
Other	.5 (1)
**Income**	
Below £5000	15.7 (31)
£5000 to £11,999	20.2 (40)
£12,000 to £15,999	13.1 (26)
£16,000 to £24,999	24.7 (49)
£25,000 to £34,999	12.6 (25)
£35,000 to £49,999	9.6 (19)
£50,000 to £74,999	3.5 (7)
£75,000 and above	.5 (1)

**Fig 1:**
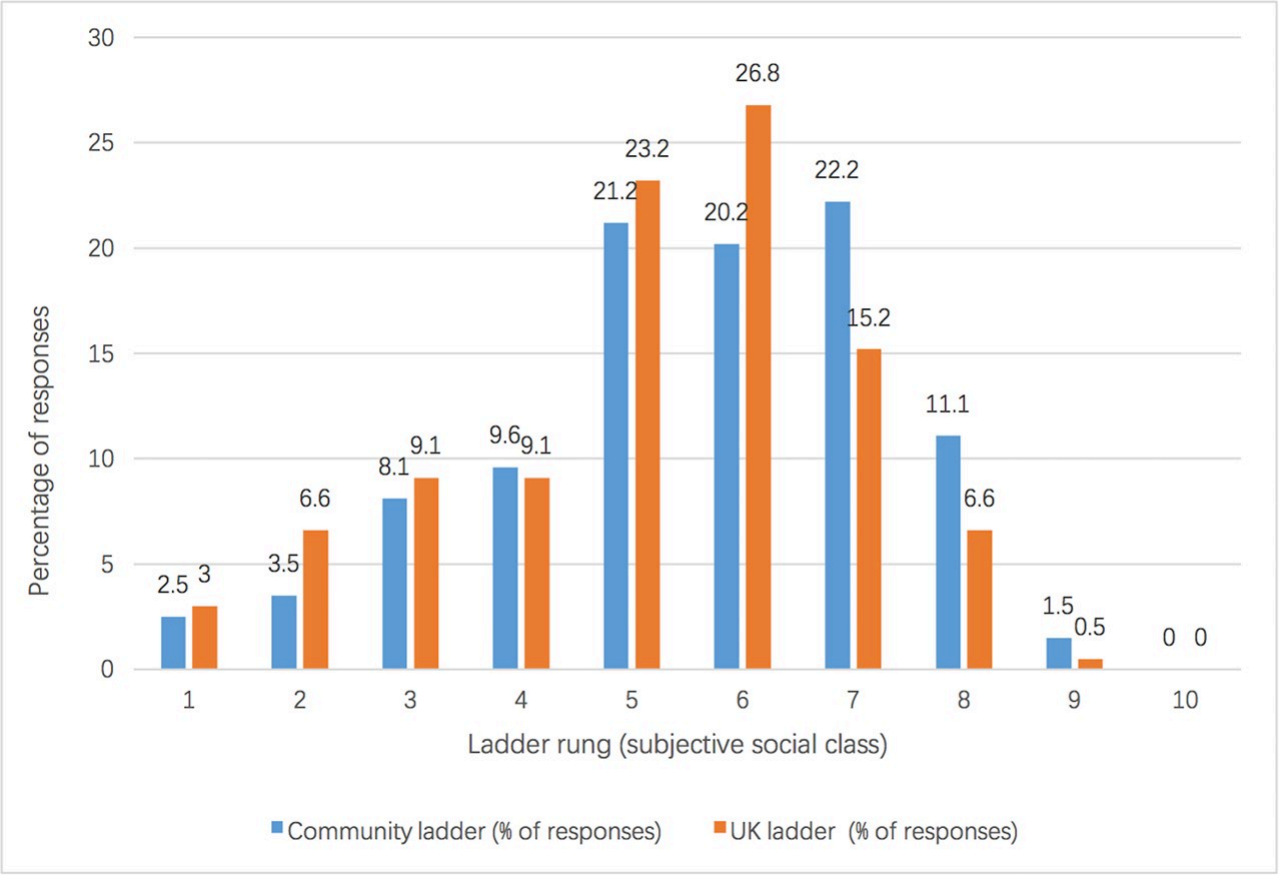
Subjective social class results of Study 1 (*N* = 198).

Subjective social class was measured by two questions on two separate but related indicators. The first was a community ladder question, in which individuals were asked about their own perspective of their social class, as represented on a ten-rung ladder (with one as the lowest level and ten as the highest). They were instructed to indicate their status within their community of friends, family, neighbors, and coworkers. The second question remained the same, but the benchmark for comparison was different: they were asked to compare themselves with the whole of the UK. Participants’ responses have been collated in Fig 1.

c Self-objectification was measured with two questionnaires. The first one was a standard self-objectification questionnaire (SOQ) [3] with a list of ten different body attributes (weight, sex appeal, physical attractiveness, firm/sculpted muscles, measurements, physical fitness level, energy level, physical coordination, strength, and health) presented in random order. Participants were asked to drag and drop the attributes to rank them from the greatest impact to the least impact each had on their physical self-concept. When scoring the responses, the ranked sum of the five attributes that were relatively less objectifying (physical fitness level, energy level, physical coordination, strength, and health) was subtracted from the ranked sum of the five attributes that were relatively more objectifying (weight, sex appeal, physical attractiveness, firm/sculpted muscles, measurements) [3]. A higher overall score represented a higher self-objectification level.
d Self-objectification was also measured through body surveillance levels using the body surveillance sub-scale from the Objectified Body Consciousness Scale (OBCS) [4]. Respondents were asked to indicate the extent of their agreement with eight statements, presented in random order on a seven-point Likert-type scale from 1 = strongly disagree to 7 = strongly agree. For contrasting statements such as *“I rarely think about how I look*,*”* raw scores were converted and awarded in reverse. We calculated a mean for all 8 items; higher scores indicated higher levels of body surveillance.

## Results

### Social class

First, we translated participants’ scores on education, income, and occupation into z-scores to combine them into objective social class scores. The variation of raw scores are displayed in Table 2.

**Table 2:** Means, standard deviations, and ranges on the measure of education, occupation, and income in Study 1.

	N	Mean	SD	Range
Education	198	3.56	1.31	7
Occupation	198	4.17	1.98	7
Income	198	3.44	1.72	7

In terms of the two indicators of subjective social class, community ladder scores and UK society ladder scores, a strong correlation was found: *r* (198) = 0.69, *p* < 0.001. Participants’ scores on the community ladder (*M* = 5.58, *SD* = 1.18) and UK society ladder (*M* = 5.20, *SD* = 1.75) were also positively associated with their objective social class, with *r* (198) = 0.39, *p* < 0.001 for the former and *r* (198) = 0.49, *p* < 0.001 for the latter. Therefore, all the above findings indicate that our measurements validly represent women’s social class in both subjective and objective ways.

### Self-objectification

The scores of the two measures of self-objectification from the SOQ (*M* = -5.60, *SD* = 13.12) and from body surveillance on OBCS (*M* = 4.29, *SD* = 1.20), were found to have a significant correlation, *r* (198) = 0.45, *p* < 0.001, This supports that our two measurements validly represent women’s objectification, and also supports the objectification theory that women who self-objectify more are also more likely to pay a greater amount of attention to, and monitor, their bodies.

### Social class, self-objectification, and body surveillance

We did not find any significant correlation between women’s objective social class and their self-objectification level (SOQ), *r* (198) = 0.04, *p* = 0.570; no correlation was detected between either their scores on the community ladder and self-objectification (SOQ), *r* (198) = 0.04, *p* = 0.601 or between scores on the UK society ladder and self-objectification, *r* (198) = 0.01, *p* = 0.879. Similar results were observed in body surveillance (OBCS): in particular, no relationship was found between women’s objective social class and body surveillance, *r* (198) = 0.004, *p* = 0.954, between their scores on the community ladder and body surveillance, *r* (198) = 0.03, *p* = 0.091, or between their scores on the UK society ladder and body surveillance, *r* (198) = 0.01, *p* = 0.907 (see Table 3). These results indicate that women’s social class is not significantly associated with either measure of self-objectification, which contradicts our first prediction.

**Table 3:** Correlations between social class, Self-objectification (SOQ), and Body Surveillance (OBCS) (*N* = 198).

	Self-objectification (SOQ)	Body Surveillance (OBCS)
Objective Social Class	.04	.004
Subjective Social Class (Community Ladder)	.04	.03
Subjective Social Class (UK Society)	.01	.01

** Significant at the 0.01 level (2-tailed)

* Significant at the 0.05 level (2-tailed)

## Discussion

We did not find a significant relationship between women’s objective social class (a combined measure of education, occupation, and income) and their self-objectification levels. Nor was there a significant relationship between women’s subjective social class (as measured through their scores on the community ladder and UK society ladder) and their self-objectification levels.

According to Objectification Theory [1], self-objectification often contributes to body dissatisfaction and a possible increased risk of extreme dieting behavior. Although prior studies have indicated a positive association between women’s social class and their body dissatisfaction and dieting behavior incidence levels, the findings of our study indicate that this association may not be extended to self-objectification. Our findings support the idea that other factors affect upper class women’s higher body dissatisfaction and dieting behavior as this may not be rooted in self-objectification. However, body dissatisfaction and dieting behavior were not measured in the current studies, and more focused research is needed. Moreover, the sample from Chen and Russo’s study [20] was taken from the US, but our study was conducted in the UK. Even though these two nations are regarded as similarly individualistic cultures [31], there still needs to be a further examination of whether any subcultures and subgroups within these two countries could have influenced these different findings.

## Study 2

### Methods

This study was approved by the Psychology Research Ethics Committee, School of Philosophy, Psychology and Language Sciences, the University of Edinburgh.

#### Participants

Participants were recruited from Prolific Academic and were paid £2 each for their participation. In total, ninety-two non-student British women participated; however, ten were excluded as they completed the online survey after it closed, leaving eighty-two women to be included in this study. The participants’ ages ranged from 20 to 61 years old (*M* = 38.65, *SD* = 11.00). The majority were white (95.1%, *N* = 78).

#### Measures

**Fig 2:**
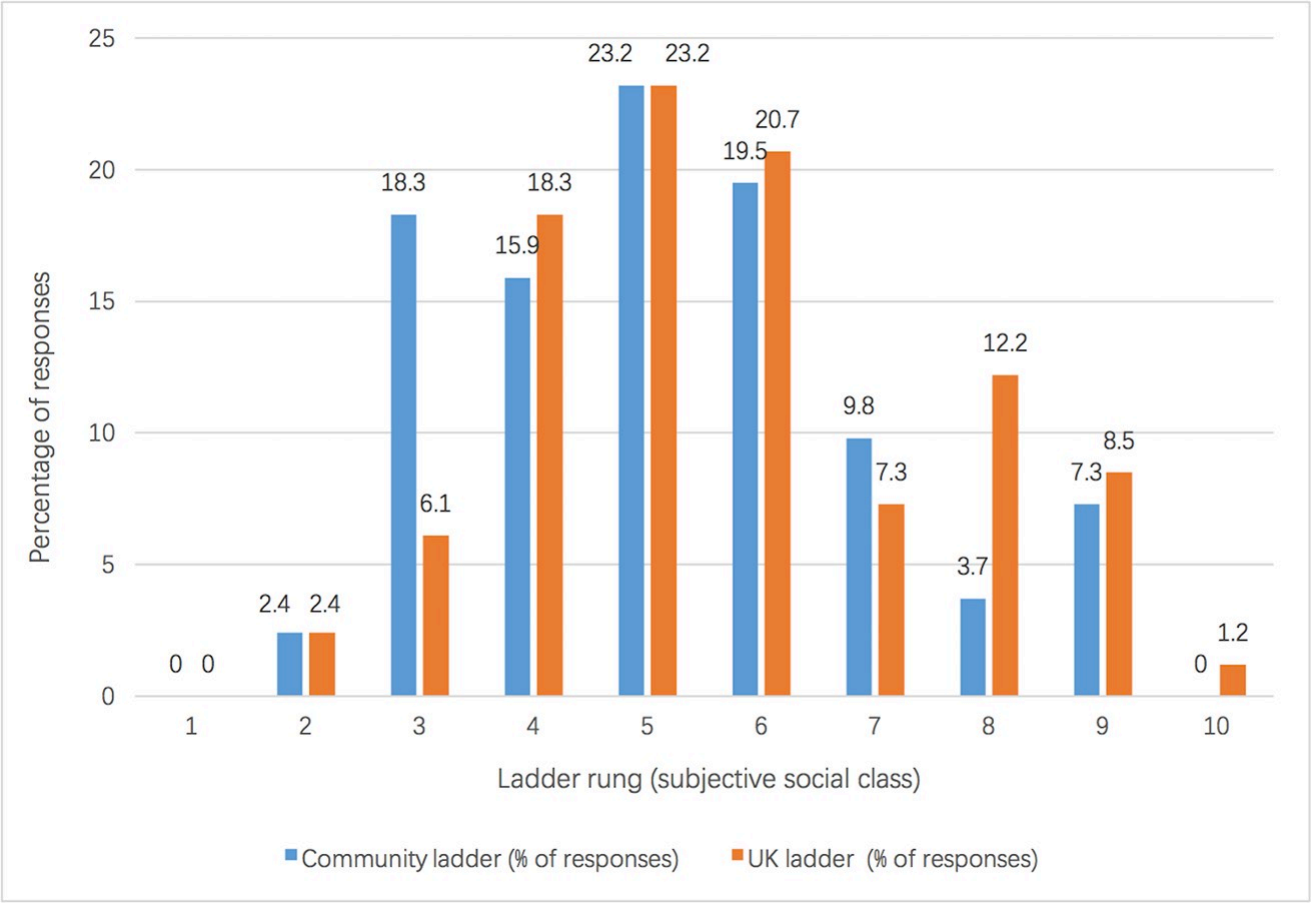
Subjective social class results of Study 2 (*N* = 82).

Each participant randomly received one of four versions of an online questionnaire comprising two parts. The first part, which included four surveys, was the same for all participants. It comprised:

- The same demographic questionnaire that was used in Study 1 to capture basic information including age, ethnicity, and marital status.
- The MSQ [29] to measure both subjective and objective social class. Objective responses on participants’ education, income, and occupation have been collated in Table 4; subjective social class was again measured using the community and UK ladders. The frequency of each ladder rung response is illustrated in Fig 2.
- The other-objectification questionnaire based on the SOQ employed in Study 1 [3] was used to measure participants’ objectification of men’s bodies. The same SOQ list of 10 different body attributes was presented in random order, but participants were instructed to rank these attributes based on their appraisal of men’s bodies instead of their own bodies (for a similar use, see [13]).
- The same other-objectification questionnaire used in (c) was this time employed with respect to other women’s bodies (for a similar use, see [13]).

**Table 4:** Social class data of sample (*N* = 82).

Variables	% (n)
**Occupation**	
Unemployed	0 (0)
Routine workers	8.5 (7)
Semi-routine workers	14.6 (12)
Lower supervisors and technicians	13.4 (11)
Small employers and own-account workers	4.9 (4)
Intermediate employees	34.1 (28)
Lower managers and professionals	19.5 (16)
Higher managers and professionals	4.9 (4)
**Education**	
Lower than high school qualification	1.2 (1)
High school/secondary school qualification	22.0 (18)
Associate degree/vocational qualification	15.9 (13)
Bachelor's degree/undergraduate Master’s degree	40.2 (33)
Postgraduate Master's degree	13.4 (11)
Doctorate	3.7 (3)
Professional (MD, JD, DDS, etc.)	1.2 (1)
Other	2.4 (2)
**Income**	
Below £5000	26.8 (22)
£5000 to £11,999	14.6 (12)
£12,000 to £15,999	12.2 (10)
£16,000 to £24,999	20.7 (17)
£25,000 to £34,999	12.2 (10)
£35,000 to £49,999	11.0 (9)
£50,000 to £74,999	1.2 (1)
£75,000 and above	1.2 (1)

To measure dehumanization, we used a standard practice (for a similar use, see [24]): a two (sexualized, non-sexualized) by two (male, female) within-subjects design using sexualization to elicit objectification. There were four different versions of the second part of the questionnaire: each version presented the participant with four different pictures from a possible set of eight, with each picture featuring an image of one of four different models, two females and two males. These pictures were selected from a freely accessible online store. In the original set of eight pictures, there were two images of each individual: one a sexualized image of the subject wearing only underwear, and one a non-sexualized image of him or her fully clothed. Each participant was given a set of four images: one non-sexualized and one sexualized female, and one non-sexualized and one sexualized male. They saw each subject only once (i.e. they were not shown a sexualized and a non-sexualized image of the same subject in a single questionnaire). The images are available from the first author on request.

Participants were then asked to respond to these images by answering two sets of questions to measure their dehumanization of the targets. The first set measured mind attribution, and was drawn from a scale devised by Piazza, Landy, and Goodwin [32] with thirteen items. Respondents were asked to rate the extent to which each target possessed one of a list of traits (e.g., *“can experience pleasure”*), presented in random order on a seven-point Likert-type scale from 1 = not at all to 7 = extremely. We then calculated a mean for all thirteen items, with lower scores indicating that the target was regarded as having less mind. The second set of nine questions, similarly presented in a random order, was adapted from a scale by Fiske, Cuddy, Glick, and Xu [33], and measured warmth, competence, and morality, with three questions for each aspect. Participants were asked to rate their levels of agreement or disagreement with a series of statements (such as *“He is intelligent”*) using a seven-point Likert-type scale from 1 = strongly disagree to 7 = strongly agree. We calculated means for each set of three items; lower scores reflected lower warmth, competence, or morality to the target.

## Results

### Subjective and objective social class

Participants’ scores on education, income, and occupation were transformed into z-scores and were added into social class scores. The variation of raw scores are displayed in Table 5. As with the findings from Study 1, the two subjective measurements (community ladder and UK society ladder scores) of social class showed a strong correlation, *r* (82) = 0.67, *p* < 0.001. Taking into account the relationship between subjective and objective measures of social class, there was a strong positive correlation between participants’ scores on the UK society ladder and their objective social class, *r* (82) = 0.50, *p* < 0.001, whereas a moderate correlation existed between the community ladder scores and objective social class, *r* (82) = 0.35, *p* < 0.05. As a result, the above findings indicate that our measures have accurately represented women’s social class in both subjective and objective ways in this study.

**Table 5:** Means, standard deviations, and ranges on the measure of education, occupation, and income in Study 2.

	N	Mean	SD	Range
Education	82	3.71	1.37	7
Occupation	82	4.20	1.75	6
Income	82	3.21	1.84	7

### Social class and objectification towards females and males

We used other-objectification questionnaires to measure women’s objectification levels towards other individuals (both women and men). We identified a strong correlation between their objectification score of women (*M* = 2.27, *SD* = 15.81) and their objectification score of men (*M* = 2.93, *SD* = 13.25), with *r* (82) = 0.68, *p* < 0.001, suggesting that women who were more likely to objectify other females also tended to objectify males. However, we did not find any significant correlation between women’s social class (subjective and objective social class) and their objectification towards either males or females (Table 6).

**Table 6:** Correlations between social class (including all indicators) and other-objectification (*N* = 82).

	Objectification of Females	Objectification of Males
Subjective Social Class (Community ladder)	.03	-.12
Subjective Social Class (UK society ladder)	.17	.03
Objective Social Class	.07	-.02

** Correlation is significant at the 0.01 level (2-tailed)

* Correlation is significant at the 0.05 level (2-tailed)

### Social class and dehumanization

The pictures of the two different target women and the two different target men were selected carefully based on similar postures, facial expressions, and clothing. As expected, entering image type as a variable in our analyses did not have a significant effect on the results. Thus, we collapsed across image type.

### Mind attribution

To examine the effects on mind attribution, we conducted a two (sexualization: sexualized, non-sexualized) by two (target gender: male, female) within-subjects ANCOVA with participants’ objective social class as a covariate. As expected based on previous research, there was a significant main effect of sexualization, such that sexualized targets (*M* = 4.75, *SD* = 0.76) were attributed less mind than non-sexualized targets (*M* = 4.84, *SD* = 0.85), *F* (1,80) = 4.55, *p* = 0.036, $\eta_{\rho }^{2}$ = 0.054. There were no other significant effects, nor was the covariate significant in any interactions, *p*s > 0.25. Thus, objective social class was unrelated to mind attribution. While considering subjective social class (community ladder score and UK society ladder score) as a covariate, we also used two (sexualized, non-sexualized) by two (male, female) within-subjects ANCOVA. We did not find a significant effect of sexualization, nor were other variables or the covariate significant in any interactions, *p*s > 0.09. In summary, contrary to our expectations, both objective and subjective social class were unrelated to participants’ perception of targets’ mind attribution.

### Morality

A two (sexualized, non-sexualized) by two (male, female) within-subjects ANCOVA was used in order to examine the effects on morality, with objective social class as a covariate. There was a significant main effect of sexualization: sexualized targets (*M* = 4.43, *SD* = 0.94) were attributed less morality than non-sexualized targets (*M* = 4.60, *SD* = 1.02). *F* (1,80) = 4.06, *p* = 0.047, $\eta_{\rho }^{2}$ = 0.048. There were no other significant effects among variables, nor was the covariate significant in any interaction, *p*s > 0.16, which indicated objective social class was unrelated to perceptions of targets’ morality. When considering subjective social class as covariate, we could not find any significant effects on any factors and interactions; nor was the covariate significant *p*s > 0.16. All these results suggest that overall social class (objective and subjective) was unrelated to perceptions of targets’ morality.

### Warmth

To investigate the effect on warmth, we carried out the same two by two within-subjects ANCOVA with objective social class as a covariate. We did not find any relationship between women’s objective social class and their perception of targets’ warmth. No significant main effect of sexualization was observed, *F* (1,80) = 0.89, *p* = 0.349, $\eta_{\rho }^{2}$ = 0.011. In addition, no other significant effects were found, nor was the covariate significant in any interaction, *p*s > 0.06. When considering subjective social class as covariate, similar results were found, *p*s > 0.05. All these results show that overall social class (objective and subjective) were unrelated to perceptions of targets’ warmth.

### Competence

The same ANCOVA method was used to analyze participants’ perceptions of targets’ competence. There was a significant main effect of sexualization, such that sexualized targets (*M* = 4.45, *SD* = 0.86) were attributed less competence than non-sexualized targets (*M* = 4.71, *SD* = 0.89), *F* (1,80) = 8.45, *p* = 0.005, $\eta_{\rho }^{2}$ = 0.096. However, there were no other significant effects, nor was the covariate significant in any interaction, *p*s > 0.24. When using subjective social class as covariate, we could not find any significant effects in any variables; nor was the covariate significant, *p*s > 0.05. The above results demonstrate that social class (objective and subjective) was unrelated to competence.

## Discussion

The aim of our second study was to investigate the relationship between women’s social class and their objectification of other women and men. As a result, we did not find either participants’ objective or subjective social class to be related to their other-objectification towards either other women or men. We also did not find a relationship between their social class and dehumanization of others in their perception of others’ mind, morality, warmth, or competence. All the above findings suggest women’s social class did not affect the degree to which they objectified (and dehumanized) others.

Although we did not find that women’s social class had any main effect on any of these variables, a significant difference was observed between participants’ perceptions of sexualized and non-sexualized targets of both genders, which supports the findings from Loughnan et al. [24]. Also consistent with the main findings from Study 1, no correlation was detected between women’s social class and their self-objectification levels, and based on the association between self- and other-objectification, this suggests that our findings are based on accurate measures, despite there being no apparent relationship between social class and other-objectification.

## General discussion

In our research, we conducted two studies to directly examine the relationship between women’s social class (objective and subjective) and their self-objectification and body surveillance, and the relationship between their social class and their other-objectification and dehumanization of men and other women. The combined results from these two studies indicated there was no observable relationship between women’s social class and objectification; thus, we inferred that women’s social class would not have an effect on how they objectify themselves or other individuals. As discussed above, these findings fill a void in existing research, making an important contribution to this field of knowledge, and thereby deepening our understanding of the relationship between women’s social class and objectification.

In our studies, we kept a heterogeneous sample to represent a broader population with considerable variance across age, marital status, education, occupation, and income; we also used multiple measures (or indicators) for each independent (subjective and objective social class) and dependent variable (self- and other-objectification). Moreover, in order to capture a more representative sample population, our study differed from Chen and Russo’s study [20], in which all participants were undergraduate students–a population more prone to social class mutability. The fact that we chose to exclude students from our study, and instead measured participants’ social class through their education, income, and occupation, could account for these different results. Our research also lays a foundation for future research, raising questions about why women from different social classes of varying education, income, and occupations, display no significant class-based differences in how they perceive themselves and their own bodies, as well as the bodies of other women and men.

A potential reason why the relationship between women’s social class and their objectification level was not observed is that individuals are likely to be exposed to media which have objectified representations of women and men (such as advertisements for beauty products and sexualized women and men in movies or on Facebook). This media environment may be highly pervasive regardless of ones’ social class. Previous research has found people’s internalization of objectified media (ideal body shape) led to body surveillance [34]. Further research is still needed to detect whether women from different social classes are exposed to and internalize objectified media differently.

Nevertheless, potential limitations arising from our study should be acknowledged, and could be addressed by further research. Both Study 1 and Study 2 were completed online to access a broader range of participants across different demographic and socio-economic backgrounds than a laboratory experiment would have afforded. However, it was difficult to control the environment in which participants responded. For instance, we were unaware of whether they were alone or surrounded by others, and of the gender of any possible companions. Moradi and Huang [2] mentioned two studies in their review of Objectification Theory: in these, individuals’ self-objectification was heightened by their interaction with language that included objectifying words [35], and by being watched by or anticipating the gaze of men [36]. This suggests that participants’ responses could potentially have been affected by heightened self-objectification resulting from their interaction with others, or the feeling of being watched while they were completing the questionnaire. Further research needs to be conducted in a laboratory setting to control external influences on participants and obviate potential confounding variables.

Moreover, these two studies were completed by two different sets of subjects. A more reliable method of measurement would be to examine the effects of social class on both self-objectification and other-objectification in the same subjects. This was not attempted in the present study because we were concerned with eliminating response-bias, such as demand characteristics [37, 38]. The most commonly used other-objectification questionnaires are drawn from the self-objectification questionnaires of Noll and Fredrickson [3], producing very similar content. We were concerned that if the same participants in our study had been presented with both questionnaires (self-objectification and other-objectification), they could have deduced the purpose of the study, with a resultant effect on their responses. To resolve the above problems, future studies could employ other types of experiments: for instance, the Implicit Association Test (IAT) could be used to measure participants’ reaction time to gauge their implicit objectification towards themselves and others. Another potential method of measurement would be a psycholinguistic study in which participants are presented with two pictures (sexualized versus non-sexualized) and asked to describe them in their own words. Their language would then be analyzed, including the frequency with which their descriptions focused on the target’s face or body. Finally, eye tracking equipment could be used to gauge the direction of their gaze on images of women and to assess whether participants focused on their body or face. All these provide interesting avenues for further study.

In conclusion, this is the first paper to systematically examine social class and objectification. Across two studies using widely employed tools, we failed to find robust evidence of class being related to objectification. Despite—or even because of—the lack of correlation shown in this study, our research indicates potential directions for future research in this area, including replicating the current study using alternative tools of measurement instead of self-reported questionnaires. Confirmation of our findings using alternative tools would provide stronger evidence that there is no significant relationship between women’s objectification level and their social class. As previous studies have found a positive correlation between women’s social class and prevalence of body dissatisfaction/dieting behavior, the lack of correlation found between objectification and social class in this study would show the need for other factors to be investigated, for instance why these behaviors are more often exhibited in upper class women. However, if the findings from future research disagree with the current studies this would suggest that when studying self-objectification, certain tools of measurement are more effective than self-reported questionnaires in eliciting accurate responses from participants.
